# A Survey on Vehicle Trajectory Prediction Procedures for Intelligent Driving

**DOI:** 10.3390/s25165129

**Published:** 2025-08-19

**Authors:** Tingjing Wang, Daiquan Xiao, Xuecai Xu, Quan Yuan

**Affiliations:** 1 School of Architectural Engineering, Zhejiang College of Construction Technology, Hangzhou 311215, China; wangtingjing2006@hotmail.com; 2 School of Civil and Hydraulic Engineering, Huazhong University of Science and Technology, Wuhan 430074, China; xiaodaiquan@hust.edu.cn; 3 State Key Laboratory of Intelligent Green Vehicle and Mobility, School of Vehicle and Mobility, Tsinghua University, Beijing 100084, China; yuanq@tsinghua.edu.cn

**Keywords:** intelligent driving, vehicle trajectory prediction, deep learning, decision-making, application scenario

## Abstract

Aimed at vehicle trajectory prediction procedures, this survey provides a comprehensive review for intelligent driving from both theoretical and practical perspectives. Vehicle trajectory prediction procedures are explained in terms of the perception layer, core technology of trajectory prediction, decision-making layer, and scenario application. In the perception layer, various sensors, visual-based perception devices, and multimodal fusion perception devices are enumerated. Additionally, the visual-based multimodal perception and pure visual perception techniques employed in the top five intelligent vehicles in China are introduced. Regarding the core technology of trajectory prediction, the methods are categorized into short-term domain and long-term domains, in which the former includes physics-based and machine learning algorithms, whereas the latter involves deep-learning and driving intention-related algorithms. Identically, the core technologies adopted in the top five intelligent vehicles are summarized. As for the decision-making layer, three main categories are summarized theoretically and practically, decision-making and planning for cooperation, super-computing and closed-loop, and real-time and optimization. As for the scenario application, open scenarios and closed scenarios are discussed in theory and practice. Finally, the research outlook on vehicle trajectory prediction is presented from data collection, trajectory prediction methods, generalization and transferability, and real-world application. The results provide some potential insights for researchers and practitioners in the vehicle trajectory prediction field, and guides future advancements in this field.

## 1. Introduction

With the rapid advancement of informatization and intelligence, the application and implementation of artificial intelligence (AI) in the automotive industry have gained significant attraction, leading to remarkable improvements in vehicle intelligence. Not only scholars, but automotive companies and IT enterprises, have increasingly focused on further advancing automotive intelligence. Empowered by continuous breakthroughs in AI technology and enhanced hardware computing capabilities, autonomous vehicle-related technologies have achieved critical breakthroughs and yielded notable developmental outcomes. The industry has witnessed the emergence of autonomous driving systems equipped with partial intelligent functions, capable of adapting to localized scenarios. By the end of 2024 there were approximately 70 manufacturers of intelligent vehicles (L2 or L2+) in China, accounting for over 40 percent of all vehicles’ revenue. Meanwhile, there were about 200 companies worldwide engaged in intelligent vehicles all over the world, with sales revenue reaching about $800 billion. Among them, BYD, Tesla, AITO (Huawei), Li Auto, and Xpeng were the top five companies in 2024 in Mainland China (Deepseek R1).

The core functions of autonomous driving systems primarily include the perception module, planning module, and control module. Among these, trajectory prediction technology within the perception module enables the prediction of the vehicle’s own trajectory or the future trajectories of nearby moving objects. The predicted trajectory is essential for the planning module, as it directly influences the vehicle’s driving behavior. To ensure safe operation, the safety system must predict changes in the vehicle’s surrounding environment in the near future and formulate proactive plans. If the vehicle’s anticipated trajectory is available, the system can make timely decisions, issue hazard warnings, or avoid potential collisions, thereby achieving safer and more efficient driving operations. Consequently, accurate trajectory prediction is critical for safe vehicle navigation and serves as a cornerstone technology for autonomous driving.

**Figure 1:**
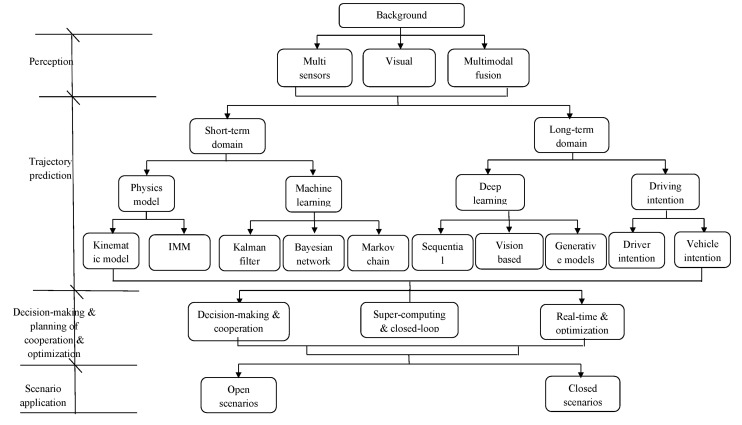
Road map of the trajectory prediction procedure.

Vehicle trajectory prediction procedures encompass the perception layer (e.g., camera, radar, sensor inputs), a trajectory prediction algorithm that forecasts potential future positions and motion trends of a vehicle based on its current movement patterns, decision-making and planning of cooperation between drivers and vehicles, optimization, and scenario application. Transmitting precise and reliable motion predictions to the decision-making and planning layer enables the system to generate rational driving strategies, significantly enhancing traffic safety. As shown in Figure 1, the trajectory prediction procedures of the intelligent vehicles focuses on the following four aspects:

(1) Perception layer: Currently, in all the intelligent vehicles, various perception devices have been installed, such as cameras, radar, sensors, bird’s eye view (BEV), etc., to collect the surrounding area data and form multimodal perception and modeling. However, the in-vehicle sensors in the perception module can only directly obtain numerical data such as vehicle speed, direction, position, and distance, which may contain inherent measurement errors. Furthermore, abstract data—such as the vehicle’s own state, the driver’s intent, or the behavior of surrounding environmental targets (e.g., pedestrians, other vehicles)—cannot be directly measured. Instead, these must be inferred through in-depth interpretation of the collected data, potentially amplifying uncertainties and errors in the system.
(2) Core technology of trajectory prediction: Most of the intelligent vehicles follow the procedure of modeling–prediction–evaluation/output as the trajectory prediction, but different vehicle models of different companies may reveal different features. The common thing is that the surrounding environmental information in vehicle trajectory prediction is dynamic and heterogeneous, so here it is divided into short-term domain and long-term domain. A vehicle’s driving intent is influenced not only by its own state but also critically depends on interactions with nearby vehicles, other moving objects, and static obstacles. Additionally, vehicle-to-vehicle (V2V) and vehicle-to-infrastructure (V2I) communication technologies can extend a vehicle’s perception range far beyond the limitations of onboard sensors. However, effectively modeling and analyzing these complex interactions remains highly challenging. In real-world scenarios, the intricacy of such conditions exacerbates the uncertainty in trajectory prediction, thereby reducing the accuracy of predicted trajectories.
(3) Decision-making and planning of cooperation and optimization: When the trajectory prediction algorithm outputs the results, real-time decision-making and takeover planning between driver and vehicle should be made immediately, and optimization is required if the decision-making is not good enough, accompanied with super-computing and closed-loop testing. Nowadays, there are still quite a few issues to be considered, e.g., takeover time, takeover process, takeover performance, takeover evaluation and optimization, the driver’s status and intent, etc. The cooperation between drivers and vehicles may highly influence the decision-making and planning of trajectory prediction, thus causing the driving risk to be increased.
(4) Scenario application: Vehicle trajectory prediction results exhibit multimodal phenomena, and some basic scenarios are similar, such as freeway or expressway, while there are some specific scenarios, for instance, in intersection scenarios, vehicles on the same road with identical historical trajectories at a given moment may exhibit divergent real-world maneuvers. This necessitates modeling and predicting trajectories based on distinct driving intents to account for potential behavioral variations. Therefore, more specific, and complicated scenarios should be constructed to reveal the real-world traffic conditions.

Consequently, the goal of this work is to conduct a survey on the state-of-the-art of vehicle trajectory prediction procedures for intelligent vehicles and provide a taxonomy of different methods, as shown in Figure 1. This study focuses on not only the algorithms or models of vehicle trajectory prediction, but also the empirical applications in different intelligent vehicle manufacturers. At last, the challenges and future research directions will be highlighted.

The contributions are as follows: The manuscript stands out by offering a holistic review of vehicle trajectory prediction procedures for intelligent driving, integrating both theoretical foundations and practical insights gleaned from collaborations with top intelligent vehicle manufacturers. It introduces a comprehensive taxonomy encompassing perception layers, core prediction technologies, decision-making frameworks, and scenario applications, thereby providing a unique and multifaceted perspective. Furthermore, it delves into specific challenges and future research directions, offering valuable guidance for the field. By bridging the gap between academia and industry, and by presenting a nuanced understanding of trajectory prediction’s complexities and opportunities, the manuscript makes a distinct contribution to the existing body of knowledge.

The outline of the paper is structed as the followings: Section 2 gives the trajectory prediction procedure from four aspects in detail, and Section 3 provides the research outlook, as well as critical and conclusive comments. In Section 4 the conclusions are reached, and limitations and future directions are described.

## 2. Vehicle Trajectory Prediction Procedures

### 2.1. Perception Layer

In intelligent vehicles, there have been various perception devices installed to collect the surrounding data. Usually, three types of perception devices are involved: sensors (e.g., millimeter wave radar, ultrasonic radar, and LiDAR (Light Detection and Ranging)), visual devices (e.g., cameras, BEV), and corresponding multimodal fusion algorithms. Choi et al. (2021) [1] collected data with LiDAR sensors and cameras to predict a machine learning-based vehicle trajectory. To overcome the low resolution and low frame rates of LiDAR, Zou et al. (2022) [2] applied LiDAR–camera fusion on the perception of autonomous driving to acquire preceding target vehicles’ trajectories. Lang et al. (2024) [3] exploited visual and context information with BEV perception and predicted the objects’ center, while Wang et al. (2024) [4] used radar point clouds and occupancy grid maps in a cross-modal manner. Zhou et al. (2025) [5] leveraged long-short term memory (LSTM) networks to predict vehicle trajectories with LiDAR data for training and testing.

In practice, among the top five intelligent vehicles, two main types of perception systems are utilized: visual-based multimodal perception and pure visual perception. Notably, Tesla employs the pure visual type, while the remaining vehicles adopt multimodal perception. For instance, BYD equips high-resolution front-facing binocular/trinocular cameras and surround-view cameras for mid-to-close-range object detection. Additionally, short-to-medium-range radars, such as Bosch 5th generation, are used for tracking the speed of vehicles and pedestrians. Ultrasonic radar aids in close-range parking scenarios, while LiDAR is only incorporated in premium models (e.g., Yangwang U8) or high-end autonomous driving versions to enhance perception redundancy in complex scenarios. By utilizing a Transformer architecture, multi-camera data are converted into a unified BEV perspective; millimeter-wave radar data are then combined with this information to model the motion intent of dynamic objects, such as crossing, lane changing, deceleration, etc. Particularly, BYD is tailored for China-specific scenarios, such as non-motorized vehicles (e.g., electric bicycles, tricycles), illegally parked vehicles, and construction barriers, while they are trained on massive datasets to improve coverage of long-tail edge cases. On the other side, Tesla adopts pure vision-based 4D environmental modeling, in which eight cameras (360° surround view (1.2-megapixel) + a front-facing triple-camera system (detection range of 250 m) + side cameras for cross-traffic capture), and no LiDAR/mm-wave radar, relying on a pure vision solution to reduce costs, with neural networks compensating for the absence of physical sensors. Four-dimensional vector space modeling is employed to convert visual data into dynamic occupancy grids (3D space + time) to predict future trajectories of obstacles (vehicles, pedestrians, unknown objects), pixel-level semantic segmentation can identify static elements (lane markings, traffic lights, construction cones) and provides dynamic updates to their status (e.g., temporary lane changes), and the HydraNets’ multi-task network helps processes tasks (object detection, depth estimation, motion prediction) in parallel, leveraging shared backbone network features to optimize computational efficiency (single-frame processing time <30 ms). More specific settings are listed in Table 1.

### 2.2. Core Technology of Trajectory Prediction

The highly dynamic nature of vehicle trajectories, complexity of scenarios, and interactions with other agents pose significant challenges for trajectory prediction. Research on vehicle trajectory prediction is essential, as it serves as a critical foundation for ensuring the safety, stability, and reliability of autonomous driving planning and decision-making. In recent years, with advancements in autonomous driving technology, research on vehicle trajectory prediction has seen a surge both domestically and internationally, and there have been various approaches and methods [6,7,8,9,10], as shown in Figure 1.

A. **Vehicle trajectory prediction of the short-term domain**

Vehicle trajectory prediction of the short-term domain refers to forecasting a vehicle’s future trajectory within a brief time horizon based on its current state. This typically involves either state prediction using short-term data or modeling vehicle dynamics and kinematics to estimate future states and trajectories. These approaches can be categorized into two main types: physics-based motion models, and machine learning models, which are often integrated in practice.

a. Physics-based models

Physics-based prediction methods primarily model vehicle motion based on dynamic or kinematic constraints to forecast future states. However, since dynamic models necessitate numerous internal vehicle parameters (such as engine torque and tire friction), these parameters are generally unobservable by external sensors. In contrast, kinematic models are simpler and more straightforward as they do not consider internal parameters or forces that may influence motion, relying instead on mathematical relationships between motion variables (e.g., velocity, acceleration, curvature) to describe vehicle behavior. Zhang et al. (2017) [11] transformed the positions of obstacle vehicles in the Internet of Vehicles (IoV) to calculate relative positions, distances, and velocities. By applying the Kalman filter (KF) and a constant velocity (CV)-based vehicle model to predict obstacle trajectories, they achieved collision warnings to enhance driving safety. Xie et al. (2018) [12] proposed an integrated vehicle trajectory prediction method called Interactive Multiple Model Trajectory Prediction (IMMTP), which combined physics-based and maneuver-based approaches. This method enables accurate short-term predictions while attempting long-term predictions with broader perspectives, demonstrating smaller prediction errors compared to traditional methods. Xiao et al. (2020) [13] introduced an interactive multiple model (IMM)-based vehicle trajectory prediction method that fuses motion and maneuver models. Initially, the CTRA motion model and unscented Kalman filter (UKF) were employed to predict uncertain future vehicle trajectories. Subsequently, a simplified maneuver recognition model leveraging historical trajectory-lane spatial–temporal relationships was used for trajectory prediction. Finally, motion and maneuver models were integrated via IMM for comprehensive trajectory prediction. While the kinematic model achieved minimal errors and high precision in short-term predictions, its errors increased significantly with extended prediction durations, deviating from actual motion trends. Geng et al. (2023) [14] proposed a physics-informed deep-learning framework by making full advantages of data-driven and physics-based models. Physics-uninformed neural networks (PUNNs) and intelligent driver models (IDMs) as physical models were constructed, and their spatiotemporal transferability and physics-informed mechanism were verified to be excellent. Similar study by Yao et al. (2023) [15] combined physics-based with learning-based models to construct a physics-aware learning-based mode for trajectory prediction of congested traffic.

b. Machine learning models

Machine learning-based vehicle trajectory prediction methods establish mathematical models by correlating historical trajectory data with predicted trajectory data. Considering that vehicle states and trajectories do not change drastically over short periods, mathematical models constructed through state estimation and probabilistic prediction of sequential data can effectively forecast trajectories.

(1) Kalman filter prediction

A KF estimates the current state of a vehicle by first predicting it based on the previous state, then fusing this predicted state with real-time measurements through weighted optimization to obtain the optimal state estimation at the current moment. This process involves two key phases: prediction (using prior state and system dynamics) and update (adjusting predictions with sensor measurements weighted by their uncertainties). The weighting mechanism balances prediction reliability and measurement accuracy, ensuring minimal estimation error. Schulz et al. (2018) [16] proposed a multi-model unscented Kalman filter (UKF) that integrated all vehicles into a unified state–space framework, enabling trajectory prediction by accounting for inter-vehicle interactions, which could benefit from airship model uncertainties and wind disturbance estimation. To address challenges in complex real-world road scenarios and diverse vehicle states, Abbas et al. (2020) [17] developed five distinct extended Kalman filter (EKF) models based on possible vehicle states, combining EKFs with the interactive multiple-model (IMM) framework for mathematical model construction and probabilistic model evaluation to estimate future vehicle positions. Lefkopoulos et al. (2020) [18] introduced a multi-vehicle motion prediction scheme using an interactive multiple-model Kalman filter (IMM-KF), integrating physics-based, maneuver-based, and interaction-aware methodologies, and resolving multi-vehicle motion estimation and prediction through a hierarchical structure encoded via priority-based vehicle hierarchies. Deng et al. (2023) [19] proposed vehicle trajectory prediction based on current statistical models and a cubature Kalman filter. This method considered the acceleration variation rules in the actual motion process, avoided unnecessary computation, and improved the prediction accuracy. The KF demonstrates stable and accurate short-term predictive capabilities when handling noise-free trajectory data, but its long-term trajectory prediction suffers from significant error accumulation. Current approaches typically employ KF for preliminary data processing to smooth observations, subsequently integrating it with other methods (e.g., neural networks or nonlinear filters) to enhance predictive performance. This hybrid strategy leverages a KF’s strength in noise suppression, while addressing its limitations in modeling complex nonlinear dynamics over extended horizons.

(2) Bayesian network prediction

A Bayesian network (BN) is a probabilistic graphical network grounded in Bayesian inference, which models vehicle states using directed acyclic graphs (DAGs), where nodes represent variables and edges encode conditional dependencies. Schreier et al. (2016) [20] proposed an integrated Bayesian framework for maneuver-based trajectory prediction and criticality assessment, utilizing Bayesian detection to identify maneuver distributions of vehicles in traffic scenarios and enabling trajectory forecasting based on inferred maneuvers. This approach leveraged the network’s conditional probability tables (CPTs) to quantify state–probability relationships, while its hierarchical structure facilitated dynamic adaptation to evolving traffic interactions. Jiang et al. (2023) [21] presented a probabilistic vehicle trajectory prediction based on a dynamic Bayesian network (DBN). A Gaussian mixture model–hidden Markov model was designed and was employed as a node in the DBN to identify the driver’s intention, which improved the prediction accuracy. Zhou et al. (2025) [5] presented a systematic method for LiDAR-based trajectory prediction, in which LSTM networks were to predict vehicle trajectories while Bayesian optimization was employed to search for optimal hyperparameter values. Bayesian networks exhibit robustness on small-scale datasets due to their probabilistic graphical structure and efficient management of conditional dependencies However, their predictive accuracy may be compromised by their reliance on assumed prior probabilities, particularly when these assumptions do not align with real-world data distributions. This limitation arises because BNs derive posterior probabilities based on prior probability assumptions and observed data, and incorrect priors can propagate errors within hierarchical reasoning frameworks.

(3) Markov prediction

A Markov model is a probabilistic transition system that relies on state transitions governed by probability distributions. The probability transition matrix it employs serves as the core mechanism determining prediction accuracy, as it mathematically defines dependencies between system states and constrains the model’s predictive capacity. Ye et al. (2016) [22] developed a vehicle trajectory prediction algorithm by exploring dual-layer hidden states in historical trajectories, proposing a dual-layer hidden-state Hidden Markov Model (HMM) framework. This method enables k-step ahead prediction of vehicle trajectories within localized spatial units. Wang et al. (2018) [23] proposed the kernel variable length Markov model (KVLMM) by combining sequence analysis with the Markov statistical model to predict trajectories. The proposed model improved the prediction accuracy and lowered the algorithm complexity. The strength of Markov models lies in their ability to calculate probabilities for systems with maintenance capabilities and multiple degradation states. However, their prediction accuracy is relatively low. First-order Markov models solely consider the influence of the current trajectory point on future points, neglecting the full utilization of historical trajectory data. Conversely, higher-order Markov models elevate computational complexity, making them impractical for training with extensive trajectory datasets. Furthermore, they exhibit sensitivity to fluctuations in vehicle trajectories and are inadequate for medium-to-long-term system predictions.

In summary, although short-term vehicle trajectory prediction methods can forecast vehicle motion states and trajectories over brief periods, their inherent limitations prevent them from resolving core challenges in trajectory prediction, necessitating the exploration of long-term prediction methods for viable solutions.

B. **Vehicle trajectory prediction of the long-term domain**

Trajectory prediction methods of the long-term domain focus more on the long-term driving trends or routes of traffic vehicles, rather than short-term motion states. The information required for traffic vehicle trajectory prediction mainly encompasses historical observation information, road features, the surrounding traffic environment, and exhibited driving behavior. Trajectory prediction methods of the long-term domain can be primarily categorized into two types: (1) using large amounts of historical trajectory data to identify motion patterns, then estimating and predicting future trajectory changes based on given trajectories and (2) combining current road information with recognized driving behavior of traffic vehicles to assess long-term driving trends, thereby predicting the long-term trajectory.

(1) Deep-learning method

Deep-learning and neural network-based prediction methods depend on the training of large-scale networks and integrate contextual information (e.g., maps, interaction information between targets) to learn more comprehensive and intricate features from historical trajectory data. These methods also account for diverse environmental factors (e.g., lane changes on highways and urban roads, roundabouts with stop/yield signs, unprotected left-turn intersections), enabling more accurate vehicle trajectory predictions. These approaches mainly include single-vehicle trajectory prediction and multi-vehicle interactive trajectory prediction. The former considers the influence of surrounding environmental information (including other vehicles) on the predicted vehicle to generate its trajectory, without predicting trajectories for other vehicles, while the latter not only models interactions between neighboring vehicles and their impact on the ego vehicle, but also predicts trajectories for all neighboring vehicles, which are then integrated to predict the trajectory of the target vehicle.

For single-vehicle trajectory prediction in deep learning, methods based on long short-term memory (LSTM) encoder–decoder architectures are currently predominant, as they effectively address gradient vanishing and explosion issues inherent in recurrent neural networks (RNNs) during long-term learning. Early research primarily utilized an LSTM encoder to capture the vehicle’s historical trajectory, extracting motion features and information about the surrounding environment. These elements were subsequently integrated for trajectory encoding, with the decoder outputting the predicted vehicle motion trajectory thereafter. As research progresses, addressing increasingly complex prediction challenges has rendered single-model approaches less effective. Consequently, hybrid model-based trajectory prediction methods are growing in prevalence, representing a key future trend. Xiao et al. (2020) [24] combined a unidirectional and bidirectional LSTM (UB-LSTM) vehicle trajectory model with behavior recognition. The identified vehicle behavior and information from a behavior recognition model were incorporated into the model to predict the horizontal and vertical speeds, as well as the coordinates of the vehicle. Similarly, Wang et al. (2020) [25] introduced a knowledge-driven LSTM neural network for predicting vehicle trajectories, where the target vehicle’s trajectory was predicted by LSTM based on the prediction reference baseline, and the predicted trajectory considered both posterior and prior knowledge. The results demonstrated that the proposed model could adapt to various driving scenarios and achieve high accuracy in predicting trajectories. Rossi et al. (2021) [26] proposed deep-learning models based on LSTM and a generative adversarial network (GAN). It was observed that LSTM-based models exhibited superior performance in unimodal scenarios, whereas generative models performed optimally in scenarios with higher multimodality. Li et al. (2022) [27] proposed a clustering convolution-LSTM (CC-LSTM) model to predict vehicle trajectories, and the spatial–temporal features were fused by the Las Vegas Wrapper (LVW) algorithm. The simulation results confirmed that the CC-LSTM prediction model could achieve real-time performance while maintaining high accuracy. Continuously, Chen et al. (2023) [28] developed a knowledge graph convolutional network LSTM (KGCN-LSTM) model to improve the accuracy and robustness of trajectory prediction. Point of interest (POI) information was considered as the prior-knowledge of trajectory by the GCN.

For trajectory prediction, relying solely on historical trajectories of surrounding vehicles is inadequate, as the extracted interaction dynamics between vehicles remain limited in scope, providing constrained effectiveness in improving autonomous vehicle trajectory forecasting. Therefore, it is crucial to consider the influence of future trajectories from both neighboring vehicles and other moving objects within the environment on the predicted path of the autonomous vehicle.

Dai et al. (2019) [29] proposed a spatiotemporal LSTM-based trajectory prediction model. Spatial interactions were embedded into LSTM model to measure the interactions between neighboring vehicles. Messaoud et al. (2020) [30] proposed a model combining grid-based trajectory encoding with LSTM and multi-head self-attention. The multi-head attention module models interactions between the target vehicle and neighboring vehicles based on their importance, while the decoder integrates interaction vectors and target vehicle encodings to generate predicted trajectory distributions. Yu et al. (2021) [31] developed an attention-enhanced LSTM trajectory prediction model by forecasting surrounding vehicles’ future trajectories and transforming their kinematic data (e.g., relative positions and velocities) into the target vehicle’s reference frame. Concurrently, they introduced a road geometry linearization methodology that enables model adaptation to diverse roadway configurations, significantly enhancing cross-environment generalizability. Sheng et al. (2022) [32] proposed a graph-based spatial-temporal convolutional network (GSTCN) to predict multimodal future trajectory distributions for all neighboring vehicles. The architecture employs graph convolutional networks (GCNs) to process spatial interactions and convolutional neural networks (CNNs) to capture temporal dependencies. The integrated spatial–temporal features are subsequently encoded and decoded through gated recurrent unit (GRU) networks, enabling probabilistic trajectory distribution generation. To interpret the influence of historical trajectories and neighboring vehicles on the target vehicle, Lin et al. (2022) [33] developed an LSTM model with spatiotemporal attention (STA) mechanisms for vehicle trajectory prediction. The spatiotemporal attention weights provided by this model enhanced its interpretability. An identical study by Jiang et al. (2022) [34] introduced a spatial–temporal attentive LSTM (STAM-LSTM) encoder–decoder model for trajectory prediction. In this model, spatial attention was employed to capture the spatial relationships among neighboring vehicles while the temporal attention was adopted to assess the effects of different historical time steps. Zheng et al. (2024) [35] constructed a hierarchical trajectory prediction method with a graph attention network (GAT) to estimate the interactions of surrounding vehicles and LSTM to predict the future trajectory of the target vehicle; a similar study by Min et al. (2024) [36] proposed a hierarchical LSTM-based model to predict vehicle trajectories, encompassing three sequential steps, driving intention prediction, lane change time prediction, and trajectory prediction. Qiao et al. (2024) [37] proposed a social-attention LSTM model to predict the future trajectories of neighboring vehicles. A social-pooling layer was utilized to capture cooperative behaviors and mutual influences between vehicles, while a self-attention mechanism was integrated to assign weights to the inputs and outputs of the social-pooling layer.

In summary, the rapid advancement of deep learning has established data-driven trajectory prediction methods as a predominant research focus. Previous studies have demonstrated that these deep-learning approaches possess fundamental capabilities in addressing the inherent limitations of conventional prediction methods for vehicular trajectory forecasting. With the continuous expansion of dataset scales, enrichment of social interaction contexts, and systematic inclusion of corner cases, experimental environments progressively approximate real-world operational conditions. By strategically integrating multi-source data inputs and architecting neural network structures with computational efficiency, substantial improvements can be achieved in both prediction accuracy (potentially exceeding 15–20% error reduction) and computational throughput (enabling sub-100 ms inference latency) for autonomous driving systems.

(2) Driving intention

The driving intention prediction method for dynamic vehicles enables the characterization of their driving behaviors over extended time horizons. Driving behavior recognition enhances comprehension and predictive capabilities in dynamic environments through long-term forecasting, thereby improving intelligent decision-making and enabling deeper situational awareness, thus holding significant importance for environmental perception. Driving behaviors can be categorized into driver behaviors and vehicle maneuvers. Driver behavior recognition focuses on predicting operator intentions through analysis of driver characteristics and traffic context, primarily concerning ego-vehicle operators. Conversely, vehicle behavior prediction analyzes traffic participants’ kinematic patterns and environmental states to anticipate their maneuvering intentions.

Chandra et al. (2020) [38] developed a rule-based behavioral prediction algorithm that categorizes traffic participants as aggressive (speeding), conservative (low-speed), or neutral through behavioral classification analysis. Liu et al. (2021) [39] constructed a framework of vehicle trajectory prediction, in which the first part built the driver characteristics and intention estimate model, while the second part established a classified Gaussian process model. Chen et al. (2022) [40] proposed a spatial–temporal dynamic attention network for vehicle trajectory prediction, and a driving intention-specific feature fusion mechanism was presented to integrate temporal and social features. Continuously, Jiang et al. (2023) [21] addressed the real-time vehicle trajectory prediction with a intention-aware interactive transformer (IIT) model, while IIT was illustrated with inter-vehicle attention and intra-vehicle intention. Li et al. (2023) [41] proposed an intention–convolution and hybrid-attention network for trajectory prediction, while an intention–convolution social pooling module was introduced to extract complete interaction about the driver’s lane-change intention and inter-vehicle interaction. The results demonstrated that the proposed model outperformed currents methods in long-term prediction. Yuan et al. (2024) [42] presented a temporal multi-task mixture of experts (TMMOE) model to predict vehicle trajectory and driving intention. Three layers were included, which shared one employed temporal convolutional network (TCN) to extract temporal features, while an expert layer incorporated the gating mechanism to filter the temporal dependence of sequences, and the fully connected layer was applied to export prediction results. Zhang et al. (2024a) [43] put forward a vehicle trajectory prediction model with a spatial–temporal dilated casual convolutional transformer network for the driving intention and spatial interaction features. Temporal information was extracted by adopting a bi-directional LSTM network, while adaptive fusion of driving intention was conducted for addressing the output of a multimodal trajectory predication module. An identical study by Zhang et al. (2024b) [44] constructed the multimodal vehicle trajectory prediction model with five components, including a driving intention fusion module. The latest work by Sun et al. (2025) [45] investigated vehicle trajectory fusion prediction based on a physical model and driving attention identification. Trajectory 1 was obtained with constant turn rate and acceleration (CTRA) model, and then a hidden Markov model (HMM) was employed to identify driving intentions to predict Trajectory 2. Both trajectories were fused into a predicted trajectory, and it was demonstrated the performance was better. Chen et al. (2025) [46] and Gao et al. (2025) [47] investigated multimodal vehicle trajectory prediction based on intention inference and an intention-enlightened decoding model, respectively.

To sum up, the vehicle trajectory prediction method based on driving intention behavior recognition can handle complex scenarios through related intention behavior recognition, exploring interactions between high-level and low-level information. It integrates intention behavior prediction with vehicle trajectory prediction while enabling online adjustment of behavior and intentions. Although this approach may exhibit lower accuracy in the initial stages of prediction, it achieves better performance over the long term.

In practical applications, among the top five intelligent vehicles, there are primarily two types of trajectory prediction models: physics-based and deep-learning-based. However, each brand may incorporate their own specific feature to these models. For instance, Li Auto integrates two types of models to predict trajectories, one is to generate baseline motion trajectories (e.g., constant-velocity linear motion or curvature-based models) based on the target’s current physical state (velocity, acceleration, heading angle), the other is to employ deep learning-enhanced prediction, which includes temporal modeling and interaction awareness. The temporal modeling analyzes the target’s historical trajectory (e.g., positional sequences over the past 3 s) to capture behavioral patterns (e.g., frequent lane changing, acceleration/deceleration habits), while interaction awareness models interactions between the target and surrounding vehicles/pedestrians via a graph neural network to predict intent (e.g., yielding, aggressive maneuvers, lane changing). There are two unique features different from other manufacturers: scene semantic understanding and multimodal probabilistic output. The former infers legally permissible paths by integrating high-definition map data (lane markings, traffic signs, traffic light states); for example, a vehicle at an intersection can only turn left or proceed straight based on these data. The latter predicts multiple possible trajectories for the target over the next 3 to 5 s, assigning a probability distribution to each (e.g., 80% probability of maintaining lane, 20% probability of lane changing) for downstream planning module decision-making. More detailed descriptions are listed in Table 2.

Based on a summary of previous studies, it is evident that short-term prediction approaches face inherent limitations, often resulting in suboptimal performance in complex scenarios due to their narrow temporal focus and inability to address critical challenges, such as sudden lane changes and multi-agent collisions. In contrast, long-term methods leverage large-scale datasets and deep-learning algorithms to integrate multi-source data (e.g., LiDAR, V2X signals, driver behavior patterns), enabling them to capture multi-agent interactions and behavioral intentions within dynamic environments.

### 2.3. Decision-Making Layer

After the perception and trajectory prediction, the vehicles and drivers need to make the decision and make some planning of cooperation; Meanwhile, with super-computing and closed loop activities, real-time behavior and optimization will be realized to continue next step for the vehicles or drivers.

The application of risk field theory in intelligent driving systems is primarily manifested in dynamically quantifying potential risks within the environment to assist vehicles in real-time decision-making and path planning. Wang et al. (2023a) [48] proposed an off-road multi-agent trajectory prediction model termed situation awareness and LSTM (SA-LSTM), which is grounded risk field theory. In the context of situation awareness extraction, risk field, and pooling layers were utilized to filter interpretable awareness, while suitable LSTM networks were chosen to accommodate the task-specific features. In a converse way, Liu et al. (2025) [49] developed a dynamic predictive driving risk field based on multi-agent trajectory prediction and digital twins system. A multiagent multimodal trajectory prediction algorithm was employed to predict future vehicle motion states, and driving risk fields can be generated by leveraging predictive multi-dimensional kinematic features. Simulation results indicated that the proposed model significantly enhanced the average pre-collision warning time, while reducing the average re-collision warning error.

Meanwhile, lane changing and behavioral decisions are part of trajectory prediction. Wang et al. (2023b) [50] came up with a decision-making model with long-term trajectory prediction for lane changing. A lane-changing decision-making model, grounded in fuzzy inference, was constructed to deduce the relative relationship between other vehicles and the self-driving car. The results demonstrated that the proposed model can ensure driving safety and enhance driving comfort. Hu et al. (2023) [51] predicted trajectories and made behavioral decisions with a joint holistic transformer network. It was found that the proposed model reduced computational costs and improved semantic relationships between scenes and agents.

Most companies have developed their own supercomputing platforms to cater to the needs of their intelligent vehicles. For instance, Tesla employs self-developed D1 chips and the ExaPOD architecture, achieving a 1.3-fold increase in training efficiency compared to GPU clusters, thereby enabling real-time iteration for models with 100 billion parameters. Meanwhile, closed-loop dynamics plays a crucial role in complex traffic scenes. Qie et al. (2024) [52] proposed a distributed decouple LSTM trajectory prediction method, and a decouple gate and a control gate were presented to characterize the closed-loop dynamics, in which the former filtered the data participating in the recurrent network, while the control gate dealt with the data outside the recurrent. The results demonstrated that the proposed method improved the trajectory prediction accuracy significantly.

**Table 2:** Core technology of trajectory prediction in the top five intelligent vehicles.

Brand	Physics Model	Deep Learning		Specific Feature	
**BYD**	**Kinematic models:** Generate baseline trajectory prediction for target vehicles/pedestrians; road topology constraints	**Temporal Modeling (LSTM/GRU):** Analyze historical trajectories (e.g., positional sequences over the past 2 s) to capture behavioral patterns	**Interaction Awareness (GNN/Attention Mechanisms):** Model dynamic interaction graphs between targets to predict group behaviors	**Multimodal Probabilistic Output:** Generate multiple plausible trajectories for the target over the next 3–5 s, annotated with confidence scores	**Scenario-Specific Optimization:** Urban intersections; highway scenarios; parking lots
**Tesla**	**Kinematic-constrained prediction**: Estimates real-time acceleration, steering angles and the curvature of dynamic targets to generate baseline motion trajectories	**Transformer attention mechanism:** Capture inter-target interactions (e.g., adjacent vehicles, lane-change intent, pedestrian crossing paths) **Game-theoretic frameworks:** Simulate human drivers’ “compete–cooperate” behaviors [53].	**Spatiotemporal joint modeling**: Integrate perception, prediction, and planning into a single neural network to directly output ego vehicle trajectories **Imitation learning optimization**: Train on millions of real-world driving datasets to learn human driver preferences	**Dynamic risk heatmap**: Map predicted trajectories into probabilistic risk fields, prioritizing avoidance of high-risk zones	**Multimodal trajectory generation**: Output six plausible trajectories for target over the next 5 s, with calculated collision probabilities
**Li Auto**	**Physics-based baseline prediction**: Generates baseline motion trajectories based on the target’s current physical state	**Temporal Modeling (LSTM/Transformer)** Analyzes the target’s historical trajectory to capture behavioral patterns	**Interaction Awareness (Social-LSTM/GNN):** Models interactions between the target and surrounding vehicles/pedestrians via Graph Neural Networks (GNNs) to predict intent	**Scene Semantic Understanding:** Infers legally permissible paths by integrating high-definition map data (lane markings, traffic signs, traffic light states)	**Multimodal Probabilistic Output:** Predicts multiple possible trajectories for the target over the next 3–5 s, assigning a probability distribution to each for downstream planning module decision-making
**AITO**	Physics-based short-term prediction: Real-time modeling of current motion states for dynamic agents to generate baseline trajectories; ideal for simple scenarios with ultra-low latency (<50 ms)	**Temporal Modeling (LSTM/Transformer):** Analyze historical trajectories from the past 3–5 s to identify behavioral patterns	**Social-GAN**: Predict multimodal trajectories for targets in complex scenarios; **Interactive game theoretic modeling**: Simulate human driver strategies in conflicting scenarios [53]	**Intersection intent recognition**: Predict target vehicle intention using turn signals, lane deviation trends, and intersection topology	**Pedestrian behavior prediction**: Infer crossing intent by analyzing posture and environmental cues **Non-motorized vehicle trajectory modeling**: Tailored to China-specific mixed traffic scenarios, and to address irregular movement patterns of e-bikes and bicycles
**Xpeng**	**Hierarchical long-short term modeling**: short-term (0–2 s) to generate high-confidence trajectories using kinematic models; long-term (2–5 s) to infer agent intentions by integrating HD map lane topology and traffic rules	**Social-LSTM/GNN**: Construct an interaction graph between dynamic agents to predict group behaviors; **Attention mechanism**: Identify key influencing factors to reduce computation load from irrelevant targets.	**Game-theory-reinforcement learning fusion**: Simulate human drivers’ compete–cooperate strategies [53];	**Spatiotemporal joint modeling**: Unify perception, prediction and planning modules into a single network to output ego trajectories. **Imitation + reinforcement learning hybrid**: Train human-like decision-making capabilities using massive real-world driving data, while optimizing safety via reward functions in simulation	**Multimodal probability output**: Produce distribution maps of future trajectories, annotated with confidence scores (e.g., 80% straight, 15% left lane change, 5% emergency braking for main-lane vehicles)

Real-time guaranteeing and optimization provide the foundation for online trajectory prediction. Based on their own computation platforms, the delay of prediction and planning paths should be less than 100 ms. For example, Li Auto deploys the algorithm on built-in computing platforms (such as NVIDIA Orin or Li Auto’s self-developed computing chips). By utilizing light weighting techniques, such as TensorRT acceleration, and asynchronous pipeline processing, it ensures that prediction latency remains below 100 ms. Zhou et al. (2025) [5] leveraged LSTM networks to predict vehicle trajectories and employed Bayesian optimization to search for optimal hyperparameter values with LiDAR. Bayesian optimization enhanced the performance and robustness of the LSTM network.

In addition to the aforementioned functions, each of the top five intelligent vehicles possesses its own unique feature for making online decisions and ensuring real-time optimization. More details can be found in Table 3.

### 2.4. Scenario Application

Currently, China’s intelligent driving technology has entered a product transition phase, with mainstream application scenarios having already begun conducting technical tests. As one of the core technologies of intelligent driving, vehicle trajectory prediction technology boasts diverse application scenarios. Meanwhile, different scenarios impose varying requirements on trajectory prediction. Based on whether the operational environment is open, these scenarios are categorized into open traffic scenarios and closed campus scenarios.

Open traffic scenarios primarily encompass autonomous public transportation (bus and taxi scenarios), unmanned road sanitation scenarios, and trunk logistics scenarios. Closed campus scenarios mainly include port transportation scenarios, unmanned campus sanitation scenarios, mining area transportation scenarios, campus logistics operations scenarios, last-mile delivery scenarios (express, food delivery, fresh goods sales), and tourist attraction sightseeing scenarios. Open traffic scenarios are primarily composed of urban arterial road scenarios [56] and expressway scenarios [57,58], while closed campus scenarios predominantly feature main road scenarios [59]. Both categories can be further subdivided into intersection scenarios (e.g., crossroads), lane-changing scenarios, and other specialized scenarios.

Vehicle trajectory prediction technology faces higher demands in open traffic scenarios due to their greater complexity and multiple influencing factors. In urban arterial road static scenarios [56], various road parameters must be considered, including traffic lights, road obstacles, traffic signs, lane markings, pedestrian crossings, and road geometry. For dynamic scenarios, it requires accounting for other moving objects such as pedestrians and vehicles. Highway scenarios [57,58] are relatively simpler, with static elements like signage and ramp intersections, while dynamic elements involve moving vehicles and lane-changing maneuvers on high-speed roads. These complex and variable environments witness frequent driving operations and recurrent traffic accidents. Implementing vehicle trajectory prediction technology enables early estimation and route planning to alleviate traffic congestion, thereby effectively improving vehicle reaction time and reducing traffic incidents.

In closed-campus scenarios, the environment is relatively simpler, primarily necessitating consideration of the vehicle’s navigation objectives (such as pedestrians and other vehicles) within the campus, and road environmental factors (like intersections and lane-changing scenarios). Characterized by low-speed, precise, and safe operations, these scenarios benefit significantly from vehicle trajectory prediction technology. By ensuring accurate navigation routes and improving operational efficiency and quality, this technology offers advantages such as cost-effectiveness, service convenience, and enhanced productivity, making it ideal for controlled campus environments.

Empirical observation indicates that among the top five intelligent vehicles, each brand possesses its own basic scenarios and specific ones, which has something in common and unique characteristics. Table 4 gives the detailed description of scenario application.

By comparing manufacturer implementations versus academic methods, there are some lessons learned:

(1) Algorithmic approaches: Comparing the algorithmic foundations of manufacturer implementations (often focused on real-world performance and scalability) with those of academic methods (which may emphasize theoretical rigor and innovation) could reveal complementary strengths and weaknesses. For example, manufacturer implementations might excel in robustness and real-time performance, while academic methods might offer more sophisticated modeling of complex interactions or driving behaviors.
(2) Data utilization: Manufacturers often have access to vast amounts of real-world driving data, which can inform and refine their trajectory prediction models. Academic approaches, on the other hand, might rely more heavily on simulated data or publicly available datasets. A comparison of how these different data sources are utilized could shed light on their respective advantages and limitations.
(3) Performance metrics: Evaluating the performance of manufacturer implementations and academic methods using a common set of metrics (e.g., prediction accuracy, computational efficiency, robustness to noise) would enable a more direct comparison. This could help to identify which approaches excel in particular aspects of trajectory prediction and where there is room for improvement.
(4) Generalizability and transferability: Manufacturer implementations are often tailored to specific vehicle models and driving scenarios, whereas academic methods might aim for broader applicability. A comparative analysis could assess the generalizability and transferability of both types of approaches, highlighting the trade-offs between specialization and versatility.
(5) Ethical and legal considerations: Manufacturer implementations and academic methods might also differ in how they address ethical and legal issues related to trajectory prediction, such as privacy concerns, liability in the event of accidents, and regulatory compliance. A comparison of these aspects could provide valuable insights for researchers and practitioners alike.

## 3. Research Outlook

At the current stage, simple trajectory prediction methods based on traditional machine learning and physics models demonstrate relatively low accuracy. To meet certain precision requirements, these methods need to be more deeply integrated with interactive perception models. With the advent of the big data era and continuous advancements in computational hardware, the availability of vast trajectory data has provided a solid foundation for research in trajectory prediction based on deep learning and neural networks, yielding some notable achievements. However, deep-learning-based vehicle trajectory prediction methods often involve large and complex models, considering intricate scenarios and numerous influencing parameters. Consequently, training speeds are slowed, and the effectiveness of predictions heavily depends on the quality of model training. When faced with complex and dynamic real-world conditions, there is an urgent need to further improve both accuracy and timeliness. On the other hand, trajectory prediction methods based on driving behavior recognition require the analysis of drivers’ behaviors and needs. Given the variability in individuals’ driving habits, this analysis can pose significant challenge. Further refinement is necessary to recognize the driving characteristics of different drivers more precisely, thereby achieving more ideal prediction accuracy.

In summary, the outlook for research on vehicle trajectory prediction methods is as follows:

(1) Multi-platform and multi-source data fusion prediction

Currently, most existing methods rely on pre-processed datasets, with only a few studies leveraging data from the Internet of Vehicles (IoV) environment, which captures information from surrounding vehicles, or solely depending on the host vehicle’s sensor systems. However, the richness of the data determines the scope of information that can be extracted. In the future, by integrating multi-source data from platforms such as the IoV, Vehicle-to-Infrastructure (V2I), and even Vehicle-to-City (V2C) networks—including vehicle location, road information, high-definition maps, roadside perception, urban data, and traffic data—models can be provided with more effective and accurate data. This will facilitate the extraction of richer and more specific features, significantly enhancing predictive foresight and substantially improving the accuracy of vehicle trajectory predictions.

(2) Lightweight framework for vehicle trajectory prediction models

To effectively deploy vehicle trajectory prediction methods in intelligent driving systems, the model framework must prioritize a lightweight design to meet the stringent requirements of embedded platforms, encompassing low computational overhead, short inference times, high real-time performance, and robust accuracy. Some key strategies for achieving this balance may include model compression and optimization, hardware–software co-design, real-time performance optimization, and testing and validation.

(3) Generalization and transferability of trajectory prediction methods

Current vehicle trajectory prediction frameworks are predominantly designed for simplified simulations or specific real-world scenarios (e.g., intersections, two-lane lane-changing). Achieving full-scenario coverage remains highly challenging. However, to achieve Level 5 (L5) autonomous driving in complex real-world environments, future research must prioritize the enhancement of the generalization and transferability of these methods. Some countermeasures may include online learning and adaptive models, cross-scenario knowledge transfer, sim-to-real, and multi-source training.

(4) Real-world application of trajectory prediction

Intelligent vehicles must operate across diverse scenarios, including urban roads, highways, and rural environments. A critical challenge lies in designing scenario-agnostic trajectory prediction models that maintain performance consistency across varying conditions. The application of trajectory prediction technology also involves legal liability and ethical concerns. For example, in the event of an accident, determining system responsibility and ensuring fairness and transparency are critical issues that need to be addressed. The deployment of trajectory prediction technology requires consideration of cost control and technical feasibility. For instance, reducing hardware and computational costs while maintaining prediction accuracy is an important research direction.

More critical and conclusive contents are extended from the outlook:

Firstly, it is crucial to acknowledge that despite the proliferation of deep-learning-based methods, the field still grapples with fundamental issues related to data quality, quantity, and diversity. Many existing models are trained on limited and often biased datasets, which hinders their generalization to real-world scenarios. This limitation underscores the need for more robust data collection and annotation strategies, as well as the development of techniques to address data imbalance and ensure representative training samples.

Secondly, the interpretability and transparency of trajectory prediction models remain a significant concern. As autonomous driving systems increasingly rely on these models for decision-making, it is imperative that their predictions are not only accurate but also comprehensible to both developers and end-users. Future research should focus on developing explainable AI methods that can provide insights into how models arrive at their predictions, thereby fostering trust and enabling better debugging and validation.

Furthermore, the integration of trajectory prediction with other critical components of autonomous driving systems, such as perception, planning, and control, requires more holistic approaches. Currently, many studies treat trajectory prediction in isolation, overlooking the intricate interplay between these components. Future work should emphasize the development of integrated systems that can leverage synergies between different modules, leading to more coherent and reliable autonomous driving behaviors.

Lastly, ethical and legal considerations surrounding the deployment of trajectory prediction technologies must not be overlooked. As these systems have the potential to impact public safety, it is essential to establish clear guidelines and regulatory frameworks that address issues related to liability, privacy, and fairness. Researchers and practitioners must engage in open dialogues with policymakers, ethicists, and the public to ensure that the benefits of autonomous driving are equitably distributed and that potential risks are mitigated.

In conclusion, while the field of vehicle trajectory prediction has made notable progress, there are still significant challenges that need to be addressed. By focusing on data quality, model interpretability, system integration, and ethical considerations, future research can pave the way for the development of safer, more reliable, and ethically sound autonomous driving technologies.

## 4. Conclusions

This paper provides a survey of vehicle trajectory prediction procedure for intelligent driving from both theoretical and practical perspectives. The four steps of vehicle trajectory prediction procedure are explained, encompassing the perception layer, core technology of trajectory prediction, decision-making layer, and scenario application. In the perception layer, various sensors and visual-based and multimodal fusion perception devices are enumerated; additionally, visual-based multimodal perception and pure visual perception employed in the top five intelligent vehicles are introduced. In the core technology of trajectory prediction, the methods are categorized into short-term and long-term domains. The former encompasses physics-based and machine learning algorithms, whereas the latter involves deep learning and driving intention-related algorithms. Similarly, the core technologies adopted in top five intelligent vehicles are summarized. In the decision-making layer, three main categories are summarized both theoretically and practically: decision-making and planning of cooperation, super-computing and closed-loop, and real-time and optimization. In the scenario application, open scenarios and closed scenarios are discussed in theory and practice. Finally, the research outlook on vehicle trajectory prediction at the current state is presented from data collection, trajectory prediction methods, generalization and transferability, and real-world application. The survey provides some potential insights for researchers and practitioners in the vehicle trajectory prediction field and orients future advancements in this field.

Some drawbacks are still available in this survey. One notable limitation is the issue of data generalization, as trajectory prediction models often struggle to maintain consistent performance across diverse and unseen scenarios. Furthermore, constraints in real-time applicability pose challenges for deploying these methods in practical autonomous driving systems, where quick and accurate predictions are crucial.

To address these limitations and advance the field, future research should focus on several key directions. Firstly, developing more generalizable trajectory prediction models that can adapt to a wider range of environments and scenarios is essential. This could involve exploring techniques such as transfer learning, domain adaptation, and the integration of multi-source data to enhance model robustness. Secondly, improving real-time applicability through the development of lightweight and efficient algorithms, as well as leveraging advanced hardware accelerators, is crucial for enabling the deployment of these models in real-world autonomous vehicles.

Additionally, future research should delve deeper into understanding and modeling the complex interactions between vehicles, pedestrians, and the environment, particularly under adverse weather conditions and varying traffic densities. This would involve not only refining existing prediction methods but also exploring novel approaches that better capture the nuances of real-world driving situations. By addressing these limitations and pursuing these research directions, the field of vehicle trajectory prediction can continue to progress, ultimately contributing to the development of safer and more efficient autonomous driving systems.

## Figures and Tables

**Table 1:** Perception settings in the top five intelligent vehicles.

Brand	Camera	Millimeter Wave Radar	Ultrasonic Radar	LiDAR	BEV	Specific Feature
** BYD **	High-resolution front-facing binocular/trinocular cameras and surround-view cameras for mid-to-close-range object detection	Bosch 5th generation for tracking the speed of vehicles and pedestrians	Close-range parking scenarios	Perception redundancy in complex scenarios	Model the motion intent of dynamic objects	Tailored for China-specific scenarios, such as non-motorized vehicles (e.g., electric bicycles, tricycles), illegally parked vehicles, and construction barriers, long-tail edge cases
** Tesla **	Eight cameras (360° surround view; 1.2-megapixel) + front-facing triple-camera system (detection range of 250 m) + side cameras for cross-traffic capture	No	No	No	Optional	4D vector space modeling, pixel-level semantic segmentation, HydraNets’ multi-task network
** Li Auto **	RGB image data for object classification and semantic segmentation	Speed and distance information to enhance moving target tracking capabilities	Optional	High-precision point cloud data for 3D object detection and distance measurement	Covered +frontal and rear wheels	Timestamp synchronization and coordinate transformation align multi-sensor data into a unified coordinate system, constructing a dynamic 4D spatiotemporal model (3D space + time series).
** AITO **	360° surround-view cameras + front-facing 8-megapixel high-definition camera, supporting long-range object detection (over 200 m)	4D imaging radar, capable of outputting target height information	Close-range obstacle detection for parking scenarios	Optional	Transformer neural network, 3D motion field model of dynamic objects	GOD network for unknown road obstacles, predicting movement trajectories
** Xpeng **	8-megapixel cameras, a 360° surround-view system with front-facing binocular cameras enabling 250-m ultra-long-range detection	5 radars fuse dynamic target speed and height information		Dual LiDARs with 120° horizontal field-of-coverage	Transformer architecture, 3D motion field for dynamic objects	Occupancy networks for unstructured obstacles; LiDAR-vision temporal alignment through cross-modal attention mechanism

**Table 3:** Decision-making and optimization in the top five intelligent vehicles.

Brand	Decision-Making and Planning	Supercomputing and Closed Loop	Real-Time and Optimization
**BYD**	**Risk field model**: Converts predicted trajectories into dynamic risk heatmaps, guiding the planning module to prioritize paths with minimal risk	**Comfort constraints:** Predicted trajectories must adhere to comfort thresholds for jerk and lateral deviation, preventing abrupt braking or sharp steering maneuvers.	**Real-time performance**: Leverages self-developed computing platforms to ensure end-to-end prediction-planning latency remains <300 ms.
**Tesla**	**Shadow mode**: Continuously compares trajectory predictions between human drivers and full self-driving, automatically triggering corner case data uploads.	**Dojo supercomputing platform**: Utilizes self-developed D1 chips and ExaPOD architecture, achieving 1.3 times faster training efficiency compared to GPU clusters, and supports real-time iteration of 100-billion parameter models.	**Simulation scenario library**: Generates tens of millions of extreme scenarios from real-world driving data (e.g., pedestrians crossing in heavy rain, multi-vehicle negotiations at intersections) to enhance long-tail scenario prediction capabilities.
**Li Auto**	**Uncertainty quantification**: Incorporates Bayesian deep learning or Monte Carlo Dropout to evaluate prediction confidence. In low-confidence scenarios (e.g., sudden target braking), the system automatically activates conservative strategies (e.g., deceleration or lateral avoidance) [54].		**Real-time computing optimization**: The algorithm is deployed on in-vehicle computing platforms (e.g., NVIDIA Orin or Li Auto’s self-developed computing chips), ensuring prediction latency below 100 ms through model light-weighting (e.g., TensorRT acceleration) and asynchronous pipeline processing.
**AITO**	**Probabilistic risk field**: Converts predicted trajectories into dynamic risk heatmaps, enabling the planning module to select optimal paths based on risk distribution (avoiding high-risk zones).	**MDC computing platform**: Huawei’s self-developed in-vehicle computing platform (e.g., MDC 810) delivers 400+ TOPS computing power, supporting multi-model parallel inference [55].	**Dynamic safety boundary adjustment:** Automatically expands safety distances and reduces confidence thresholds for lane-changing decisions in low-visibility conditions (e.g., rain/fog) or occluded object scenarios (e.g., sudden pedestrian appearances).
**Xpeng**	**Shadow Mode**: Real-time collection of trajectory discrepancy data between human driving and system predictions, automatically triggering data uploads for corner cases (e.g., aggressive cut-ins, unusual obstacles).	**Simulation testing**: Constructs a high-fidelity virtual scenario library using Unreal Engine 5 (e.g., intersections in heavy rain at night with no streetlights), covering tens of thousands of long-tail scenarios to accelerate prediction model iteration.	**Miles per intervention (MPI) optimization:** Leverages cloud-based training (Alibaba Cloud + XPeng’s in-house computing infrastructure) to continuously reduce intervention frequency for urban NGP. In 2023, MPI for urban scenarios improved to 200+ km per intervention.

**Table 4:** Scenario application in the top five intelligent vehicles.

Brand	Basic Scenario	Specific Scenario	Unique Feature
**BYD**	In scenarios like highway cruising and traffic jam following, trajectory prediction demonstrates high accuracy with human-like decision-making (e.g., moderately tolerating lane-cutting by adjacent vehicles)	Prediction capabilities remain weak for extreme cases (e.g., temporary detours in construction zones or sudden animal intrusions), necessitating driver intervention	ADAS features in mid-to-low-end models prioritize meeting basic L2-level requirements, while advanced urban NOA (Navigate on Autopilot) is reserved for premium models like the Denza N7 and Yangwang U8
**Tesla**	**Unprotected left turn**: Predicts the time window for oncoming straight traffic to pass, dynamically adjusts decisions based on the host vehicle’s acceleration capability, achieving a success rate exceeding 90%. **Highway ramp merging**: Analyzes adjacent vehicles’ steering angles and acceleration changes to assess yielding intent, enabling adaptive selection of aggressive or conservative merging strategies.	**Sudden pedestrian crossing:** Detects abrupt directional shifts or speed changes in pedestrian gait (e.g., sudden backtracking) to trigger lateral evasion maneuvers or emergency braking.	**Construction zone navigation:** Identifies temporary obstacles via occupancy grid networks, predicts construction vehicle movement paths, and plans safe detour trajectories.
**Li Auto**	**Highway scenarios:** Detects lane-cutting intent from adjacent vehicles (e.g., delayed lane changes after turn signals), adjusting the host vehicle’s following distance to ensure safety.	**Urban roads:** Predicts pedestrian crossings and sudden blind-spot intrusions by e-bikes (e.g., “sudden blind-spot intrusions” scenarios), proactively planning evasion trajectories.	**Parking lots:** Anticipates movement paths of pedestrians near parking spaces, mitigating collision risks caused by blind zones.
**AITO**	**Unprotected left turn:** Predicts the time window for oncoming straight vehicles to pass, dynamically decides to proceed assertively or yield based on the host vehicle’s acceleration capability.	**Sudden intrusion pedestrians:** Analyzes shadows from roadside obstructions (e.g., buses, greenery) to preemptively detect pedestrians crossing unexpectedly, triggering automatic emergency braking (AEB).	**Forced lane-cutting in traffic jams:** Detects adjacent vehicles’ steering angles and speed variation trends, proactively fine-tunes the host vehicle’s speed to prevent forced cuts or side-swipe incidents.
**Xpeng**	**Urban intersection left turn**: Predicts the time window for oncoming straight vehicles to pass and dynamically decides to proceed assertively or yield based on the host vehicle’s acceleration capability, achieving a success rate exceeding 95%.	**Forced lane-cutting in congested traffic**: Analyzes adjacent vehicles’ steering angles and acceleration changes to predict lane-cutting intent 0.5 s in advance, proactively fine-tuning speed to prevent being cut off. **Mixed Pedestrian and Non-Motorized Traffic**: Detects e-bike wrong-way riding and pedestrian “hesitant gait”, predicts sudden trajectory changes (e.g., abrupt backtracking), and triggers lateral evasion maneuvers.	**Highway construction zones**: Integrates temporary updates from high-definition maps to predict irregular trajectories of construction vehicles, enabling early lane changes to a safe path.
